# A Multidisciplinary Model of Dementia Care in an Underserved Retirement Community, Made Possible by Telemedicine

**DOI:** 10.3389/fneur.2016.00225

**Published:** 2016-12-23

**Authors:** Jason V. Tso, Roxanna Farinpour, Helena C. Chui, Collin Y. Liu

**Affiliations:** ^1^Keck School of Medicine of the University of Southern California, Los Angeles, CA, USA; ^2^Department of Neurology, Keck School of Medicine of the University of Southern California, Los Angeles, CA, USA

**Keywords:** teleneurology, telemedicine, dementia, Alzheimer’s, remote assessment

## Abstract

The need for memory specialists is increasing as the incidence of dementia rapidly rises across the globe. In rural areas, demand for these specialists far outstrips supply. It is increasingly difficulty for patients to receive care in a timely manner. In this paper, we document our experience using videoconference telemedicine to bring a multidisciplinary model of care to a rural retirement community in Southern California. To our knowledge, we are one of the first to integrate telemedicine into dementia care on this large a scale. Given the relatively remote location, patients and neurologists have previously had to travel great distances and bear with long wait times. With neurological consultation by telemedicine and a local team consisting of a geriatrician, a neuropsychologist, and a case manager, we have been able to provide comprehensive dementia care in this underserved area, comparable to university-affiliated California Alzheimer’s Disease Centers, typically found only in major metropolitan areas. We have shown that telemedicine can be very effective in improving access and quality of dementia care.

## Introduction

Telemedicine, the use of telecommunication and information technology in clinical evaluation and treatment, has existed in various forms for over 100 years. Its evolution is intricately linked to the advancement of information technology (1, 2). Interest in telemedicine has grown rapidly with the proliferation of high-speed internet and videoconferencing equipment since the 1990s. Many medical specialties have explored the use of telemedicine in direct patient care, as a tool to improve access and efficiency. This trend is fueled by Medicare’s decision in 1996 to allow billing for “care by telemedicine” (3).

Psychiatrists were among the first to widely adopt video-based telemedicine by initially capturing video to send to providers in what is known as “store-and forward” telemedicine (2). This advanced to include real-time video interaction, now being used by various medical specialties to provide care to underserved populations (4–6). Notably, medical subspecialties such as pulmonology and endocrinology have been using video consultation to increase treatment compliance and improve patient autonomy and outcomes (7–11).

In neurology, telemedicine has found wide acceptance in the area of stroke management. In 1999, Levine and Gorman proposed the use of telemedicine in the management of acute stroke and called it “telestroke” (12). Since then, telestroke has led the way for other subspecialties in neurology to utilize telemedicine. There is a growing interest in teleneurology as physicians are beginning to apply it to the treatment of movement disorders and multiple sclerosis (13–16).

This paper summarizes the current state of telemedicine in dementia care and documents our experience in using telemedicine to bring a multidisciplinary model of care to the Coachella Valley, a retirement community in Southern California.

## Telemedicine in Dementia Care

### Prevalence and Incidence of Dementia

As life-expectancy rises across the globe, dementia has become a major public health concern. The number of people who are 65 years and older is expected to increase from 500 million globally to one billion by 2030 (17). Three to 11 percent of people older than 65 and half of people older than 85 have dementia (18–20). Alzheimer’s disease (AD) is the most frequent cause of dementia and currently affects more than 5.4 million people in the US (21). This number is expected to be more than triple to over 16 million by 2050 as the baby boomer generation ages (22).

### Importance of Access to Specialists

Although there is no effective therapy to stop the progression of AD, timely diagnosis might help lessen early anxiety and frustration associated with cognitive decline. For some patients, pharmacotherapy might temporarily stabilize cognitive decline and behavioral symptoms. Most importantly, early recognition of disease also allows patients and families to better plan for the future and makes informed decisions regarding their medical care and quality of life.

Primary care physicians are often the first and only point of contact for older adults with memory concerns. Unfortunately, dementia remains undiagnosed by these providers in an estimated 50–67% of patients over 65 (23–27). In the primary care setting, widely used screening measures are often not sufficient to detect signs of early dementia, resulting in missed or delayed diagnosis. Even if cognitive decline is suspected, referral to a memory clinic typically takes several months and often requires long-distance travel. In addition, patients and families might put off clinical evaluation because of the social stigma associated with cognitive decline and the belief that treatment is ineffective (28, 29). Improving access to memory specialists by telemedicine, especially in underserved communities, can relieve logistic barriers and help overcome reservations about seeking timely care.

### Validity and Acceptance of Telemedicine for Cognitive Assessment

Cognitive assessment with video technology has been documented as early as 1964 (1). Comparative studies have found agreement between face-to-face (FTF) and telemedicine assessments using the Mini-Mental State Examination (MMSE), Geriatric Depression Scale (GDS), Katz assessment of Activities of Daily Living, Instrumental ADL assessment, and Informant Questionnaire for Cognitive Decline in the Elderly (30, 31). Mild cognitive impairment (MCI) and AD can be diagnosed by telemedicine as studies have found that the accuracy of diagnosis *via* telemedicine was not inferior to FTF assessment (32, 33).

In addition to the growing body of evidence validating the use of telemedicine for diagnosis, evidence is also accumulating for patient acceptance of videoconferencing in lieu of a FTF encounter. Studies have found a 98% patient satisfaction with some patients even preferring telemedicine consult due to convenience (34, 35). One large Canadian study found that even in elderly populations, a majority of patients surveyed were interested in using videoconferencing and email to manage their chronic diseases, with most noting a concern for saving time and money (36).

### The University of Southern California (USC)–Eisenhower Medical Center (EMC) Memory Assessment Clinic Experience

The Memory Assessment Center (MAC) opened in November of 2007 on the EMC campus in Rancho Mirage, California. In late 2012, an institutional agreement was made between EMC and the USC to develop a comprehensive and multidisciplinary model of care with referrals from a network of local primary care physicians and general neurologists and case management resources from the local chapter of the Alzheimer’s Association. The aims of the new model are to provide (1) timely diagnosis; (2) comprehensive management from diagnosis to social service referrals; and (3) accurate and up-to-date information regarding memory loss to the patients, their families, and the community. Below, we detail the demographics of the targeted service area, the new service model, and initial statistics.

### Patient Demographics

The Coachella Valley is located in Riverside County, one of the fastest growing counties in the US and the fourth largest county in the state of California. The Coachella Valley’s population grew by 40% from 1990 to 2000, by another 38% from 2000 to 2007, and is expected to grow an additional 30% from 2008 to 2016. Total population as of 2010 is 423,000. The county consists of eight regions with seniors disproportionately represented in the Western region.

As Table 1 highlights, the median age of residents in the Coachella Valley is higher than that in California or the nation. It has also increased at a faster rate from 2000 to 2010. Specifically, the percentage of population greater or equal to age 80 in the Coachella Valley is about twice as high as that in California or the nation.

**Table 1:** **Age Demographics from U.S. Census (37)**.

Location	2000 Median age	2010 Median age	2010 ≥60 years (%)	2010 ≥70 years (%)	2010 ≥80 years (%)
US	35.3	37.2	19	9	4
California	33.3	35.2	16	8	3
Coachella Valley	42.6	47.6	33	18	7

The Alzheimer’s Association has predicted that the number of people living with AD in Riverside County will double by 2030. Among baby boomers in Riverside who reach the age of 55, one in six will develop dementia and one in eight with AD. These statistics have led the Alzheimer’s Association to call for improvement in community-based health services and to specifically address the need for a comprehensive memory assessments center in the Coachella Valley.

## The New MAC Model

**Figure 1:**
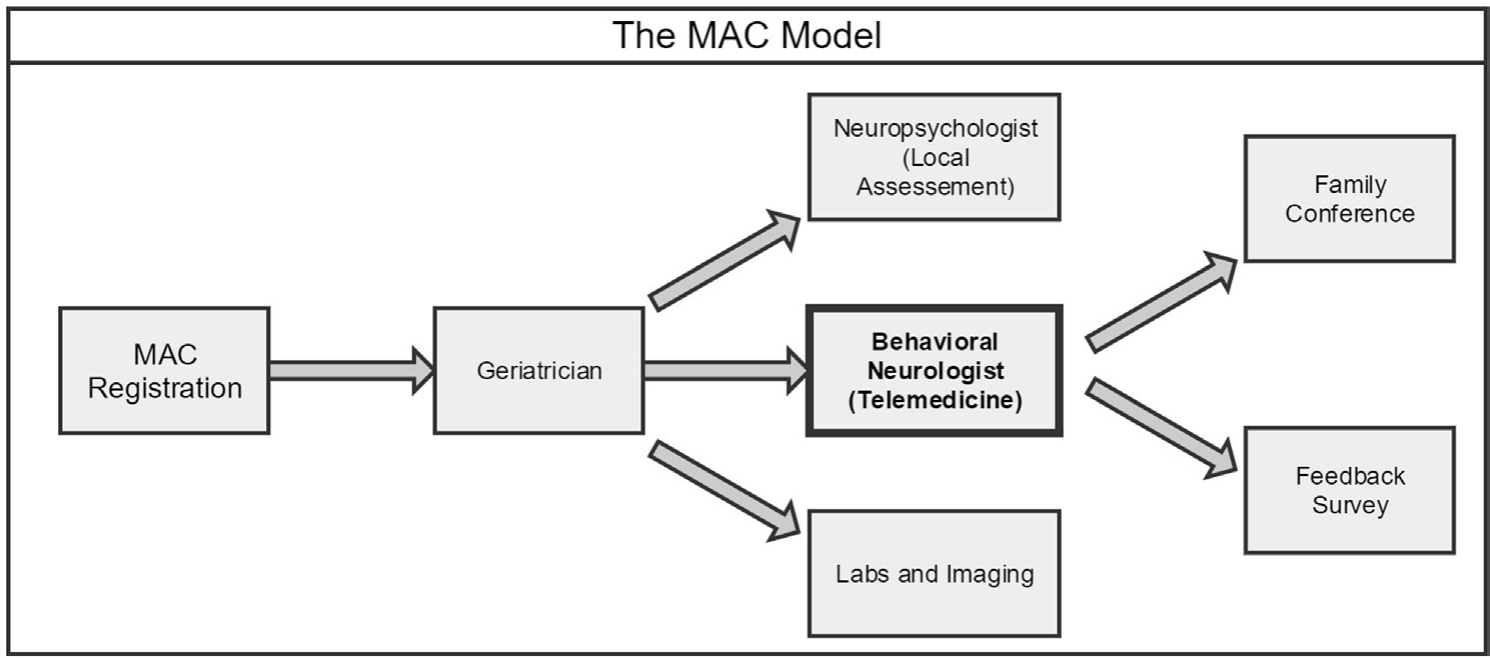
**Workflow of the New Memory Assessment Center (MAC) model: behavioral neurologist by telemedicine**.

Patients are typically referred to the MAC by local primary care physicians, general neurologists, or the Alzheimer’s Association. A small number of them come without referral, having learned about the MAC through local community outreach and publication. The team of specialists consists of a geriatrician, a neuropsychologist, a behavioral neurologist, and a case manager to fully address the needs of patients and their families. In this manner, patients receive the entirety of their care locally from diagnosis to treatment to social and support services. Central to this new model is access to behavioral neurology by telemedicine (Figure 1).

Patients are first registered by phone or in person where relevant demographic, financial, medical, and cognitive/behavioral information is gathered and entered into an electronic database. In the first visit, they meet with a geriatrician, who performs screening tests for cognition and mood, conducts a general physical and neurological exam, orders imaging and labs (CT, MRI, blood, etc.), and manages existing medical problems. The patient then sees a local neuropsychologist who assesses mental status and orientation, attention and concentration, short- and long-term memory, executive function, and depression/anxiety. All information is then shared with the behavioral neurologist on EMR by remote access for diagnosis and management by telemedicine. Using high-quality audio–video equipment, the neurologist takes additional history and performs focused cognitive and neurological assessments with the help of an onsite licensed vocational nurse (LVN). The management plan is then formulated and discussed promptly at the end of the telemedicine session, or if appropriate, in a subsequent family teleconference attended by the patient, family members, geriatrician, and case manager. The nurse works closely with the front office staff and the specialists to facilitate appointments, orders, and communication.

## Integration of Telemedicine

Telemedicine has not been widely used as part of dementia care due to the intricate nature of the auditory and visual interaction between a patient and an examiner. However, with the availability of high-quality videoconferencing technology and secure high-speed internet, this is rapidly changing.

Prior to 2013, the MAC was based on the conventional model of care. A neurologist from USC commuted every weekend from Los Angeles to Palm Desert (120 miles, 2 h each way) to assess and manage patients. Access to the neurologist was the limiting step in patient enrollment. Over time, it became apparent that this setup was not sustainable as clinic wait-time lengthened to 6+ months, and the demands of frequent travel weighed heavily on the neurologist.

In 2013, with support from benefactors, EMC, USC, and the Alzheimer’s Association, we developed a new multidisciplinary model with integration of telemedicine. A key feature of the new service model is the use of telemedicine technology in cognitive and behavioral examination. In utilizing telemedicine, specialist care is made available to resource-scarce region. To our knowledge, we are one of the first to integrate telemedicine into dementia care on this large a scale.

In our experience over the past 3 years, telemedicine poses no barrier to accurate evaluation and is as effective as a meeting in person. Accurate diagnosis in memory clinic requires detailed history from patients and families and demonstration of cognitive deficits and neurological findings in the office. With reliable and high-fidelity audio/video equipment, we have been able to obtain history and perform cognitive exam in the same manner as we would in person, with only occasional repetition of questions by the onsite staff. For neurological exam, the neurologist can rely on exam results from the initial visit with the geriatrician and perform a focused exam to demonstrate signs of cerebral degeneration, such as frontal release signs. When these signs cannot be demonstrated visually, such as the sign of cog-wheeling in arm/wrist, our nurse is trained to report tactile findings. After diagnoses are made, treatment and management locally by primary care physicians and case managers make the experience seamless for the patients and their families. Access to a behavioral neurologist is no longer the limiting factor in dementia care.

## Patient Flow and Distribution

**Figure 2:**
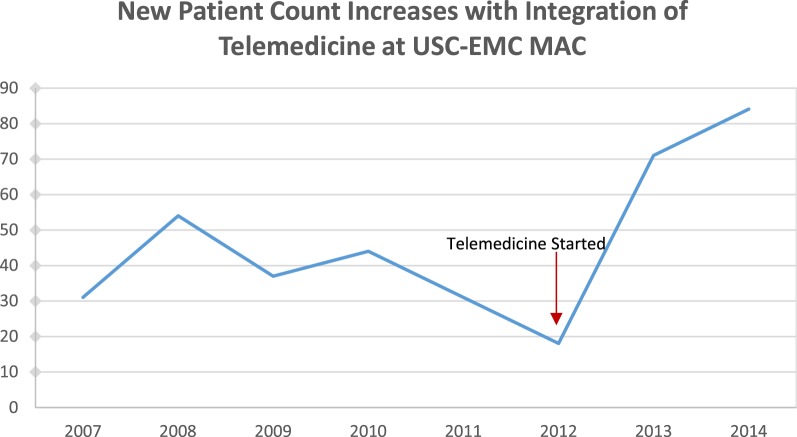
**Yearly new patient count before and after addition of telemedicine**.

From 2008 to 2012, there was a significant drop in number of new appointments, reflecting the failings of the conventional program (e.g., difficult 120-mile commute for the neurologist and inconsistent staffing by nurses on weekends) (Figure 2). With increased access to behavioral neurology by telemedicine, new patient count grew significantly in 2013 and 2014 with a twofold to threefold increase compared to previous years. The number of new patients is expected to plateau around 90 per year. Follow-up appointments for repeat cognitive exam and assessment of care-level and support total about 250 per year. The MAC (geriatric and neurology clinics combined) typically accepts 3–4 patients per day (0.5 h for follow-up and 1.5 h for new patient), 2–3 days per week. In both conventional and telemedicine models, only one neurologist attended the clinic. With telemedicine, we were able to increase the neurologist’s clinic time twofold to meet the demand. Without the long commute, the neurologist was able to spend more time on patient care. This volume of patients at the MAC is comparable to a university-affiliated California Alzheimer’s Disease Center (CADC) program.

The geographic distribution of the patients suggests that most are from the local area: 56% live less than 10 miles from EMC; 89% are under 20 miles; 8% are from further desert regions up to 180 miles away; 1% are from Northern California; and 2% are from out of state.

## Telemedicine Setup

The telemedicine sessions are staffed by a neurologist and an onsite nurse (LVN). They take place in a modified exam room in the MAC, next to the office of the geriatrician. A camera with remote-controlled zooming and turning (Lifesize Team 220 video conference codec) is installed on the wall with view of the entire exam room. Two computer monitors provide simultaneously videostreaming of the neurologist and the visual tools for cognitive examination. Fifteen minutes before each appointment, the neurologist at USC and the nurse at EMC test the audio–video system for optimal setting. For the appointment, the patient and their families are greeted and brought in by the nurse, who stays in the room during the entire session. Detailed history, focused neurological assessment, and cognitive exam are performed with the nurse’s assistance. Prior in-person instruction by the neurologist ensures that the nurse can appropriately follow instruction and report finding in a neurological exam. No special training in telemedicine is required for the neurologist.

## Investment and Billing

Setup of the telemedicine clinic requires purchase of videoconferencing equipment and training of qualified nurses to assist with the cognitive and neurological assessment. The cost of the equipment can range from a few thousand to tens of thousands of dollars depending on the availability of reliable and secure internet connection. At the MAC, equipment investment at EMC and USC added up to about $10,000. The professional fee for the telemedicine session is billed by the behavioral neurologist at USC, and technical fee is billed by EMC. Professional billing for telemedicine conducted through USC Care, Inc. was reimbursed similarly to FTF meeting at USC. Insurance reimbursement is comparable to an university-affiliated CADC, with 45% by private insurance, 50% by Medicare, and 5% by self-pay or scholarship.

## Patient Satisfaction

Satisfaction data were collected by phone calls from 46% of the patients and/or their families (*n* = 33), a number similar to rates in comparable literature (38). Patients were asked to rate various aspects of the MAC on a 5 point Likert scale. The ratings have been overwhelmingly positive with overall satisfaction with the clinic at 4.84 out of 5. General satisfaction with the neurologist was 4.88 out of 5. Satisfaction with the telemedicine system was 4.65 out of 5. Just one respondent expressed a strong dislike of telemedicine technology. 96% of those surveyed indicated a willingness to recommend the MAC to friends and family. Many patients responded that they were initially reluctant to participate in a video interview given their hearing loss, but this was a non-issue with high-fidelity videoconferencing equipment. Although most patients still prefer seeing the doctor in person, they overwhelmingly prefer the convenience of telemedicine over long-distance travel or lengthy wait-time.

## Conclusion

Telemedicine is a powerful tool to address the problem of access to care across medical specialties. In the USC–EMC MAC experience, we have demonstrated that comprehensive and seamless integration of telemedicine into dementia care is not only possible but also preferred by patient over long-distance travels. This model also allows for a solid relationship between providers that may be lost in a more traditional referral based model. The integration of telemedicine has effectively increased accessibility of behavioral neurologists to rural retirement communities of the Coachella Valley in Southern California. In the future, telemedicine is expected to become pervasive. The USC–EMC MAC serves as an efficient model for integration of telemedicine, allowing an underserved group of patients to experience multidisciplinary care within a single institution.

## Author Contributions

JT, RF, and CL: drafting of manuscript, and acquisition and interpretation of data. HC and CL: critical revision of manuscript, and conception.

## Conflict of Interest Statement

The authors declare that the research was conducted in the absence of any commercial or financial relationships that could be construed as a potential conflict of interest.
